# Association of the oxidative balance score with sarcopenia among young and middle-aged adults: findings from NHANES 2011–2018

**DOI:** 10.3389/fnut.2024.1397429

**Published:** 2024-06-04

**Authors:** Zhi Cai, Dantong Dong

**Affiliations:** ^1^School of Health Management, China Medical University, Shenyang, Liaoning, China; ^2^Center for Reproductive Medicine, The Second Affiliated Hospital of Soochow University, Suzhou, Jiangsu, China

**Keywords:** sarcopenia, oxidative balance score, NHANES, nutrition, lifestyle

## Abstract

**Background:**

Sarcopenia is associated with oxidative stress. The Oxidative Balance Score (OBS) is commonly used to assess dietary and lifestyle exposure to oxidative stress. However, few studies in the literature have assessed the correlation between sarcopenia and OBS.

**Aim:**

This study aimed to assess the association between OBS and sarcopenia among young and middle-aged adults in the United States using data from the National Health and Nutrition Examination Survey (NHANES).

**Method:**

Weighted logistic regression analysis was used to investigate the association between OBS and sarcopenia based on data from NHANES 2011–2018. Subgroup analyses were performed to observe the consistency of the outcomes, and the stability of the results was tested using sensitivity analyses.

**Result:**

The final sample included 5,525 young and middle-aged American adults. A higher OBS was associated with a lower risk of sarcopenia. In the fully adjusted model, the second (odds ratio [OR]: 0.62, 95% confidence interval [CI]: 0.41, 0.94; *p* = 0.023), third (OR: 0.50; 95% CI: 0.34, 0.74; *p* < 0.001), and highest quartiles (OR: 0.18; 95% CI: 0.11, 0.28; *p* < 0.001) of the OBS were associated with higher risks of sarcopenia when compared to the lowest quartile. This association was consistent across both dietary and lifestyle OBS scores. Our subgroup analysis revealed no significant differences between the subgroups of variables included in the interaction analysis. Sensitivity analyses revealed similar results.

**Conclusion:**

Our study showed that higher antioxidant and lower antioxidant exposure may decrease the risk of developing sarcopenia. Higher OBS scores may indicate greater protection against sarcopenia; however, further clinical studies are warranted to confirm these findings.

## 1 Introduction

Sarcopenia, a disorder related to the progressive loss of muscle mass and function, has adverse effects on falls, functional decline, frailty, and mortality (1). Recently, sarcopenia has been found in the younger population, despite its general association with advanced aging (2, 3). Globally, the number of patients with sarcopenia is expected to increase from 50 to 200 million over the next 40 years (4). Among the population aged < 60 years, the incidence of sarcopenia ranges between 8% and 36% (5). Healthcare costs can be substantially increased due to sarcopenia (6). For sarcopenia patients aged < 65 years, hospitalization costs increase even further than they do for those aged >65 years (7).

Sarcopenia can be classified into two types: primary and secondary. With aging, the aggravation of muscle fiber loss, neurodegeneration, and dysfunctional proteins in the skeletal muscles contribute to primary sarcopenia (8). Several factors can contribute to secondary sarcopenia—including insulin resistance, inactivity, poor nutrition, neuromuscular diseases, and hormonal imbalances (9). The histopathological characteristics of the two types of sarcopenia are similar, such as muscle fiber replacement by adipose tissue, increased fibrosis, oxidative stress, altered muscle metabolism, and neuromuscular junction degeneration (10). Oxidative stress refers to an imbalance between the production of reactive oxygen species (ROS), also known as free radicals, and the antioxidant defense system (11). ROS accumulation in sarcopenia occurs as a result of mitochondrial dysfunction. This accumulation can affect the function of myofibrils, motor neurons, and the sarcoplasmic reticulum—thus hindering muscle regeneration (12, 13). Therefore, a potential therapy for sarcopenia patients is to reduce ROS levels via antioxidant treatment. This treatment can include increasing dietary antioxidant intake, increasing physical activity, avoiding obesity, and avoiding tobacco smoking.

However, a single factor is unlikely to have a significant impact on the overall antioxidant/oxidative system. To evaluate the roles of multiple diets and lifestyles on the overall oxidative/antioxidant system, the Oxidative Balance Score (OBS) has been developed to measure one's general exposure to oxidants and antioxidants (14). Studies have demonstrated that a higher OBS reflects increased protection against a range of diseases—including kidney disease (15), diabetes (16), non-alcoholic fatty liver disease (17), and even depression (18). However, few studies in the literature have reviewed the correlation between sarcopenia and OBS. Therefore, this study aimed to assess the association between OBS and sarcopenia among young and middle-aged adults in the United States, using National Health and Nutrition Examination Survey (NHANES) data, for aiding the development of new preventive interventions for the condition.

## 2 Materials and methods

### 2.1 Study population

The NHANES is a cross-sectional survey aimed at assessing the health and nutritional statuses of adults and children in the United States. We included population data from the nationally representative NHANES database from 2011 to 2018. During all four cycles, we collected complete information concerning sarcopenia and OBS in adults aged >20 years. Written informed consent was obtained from each participant on joining the NHANES, and the study was approved by the ethics review board of the National Center for Health Statistics.

**Figure 1:**
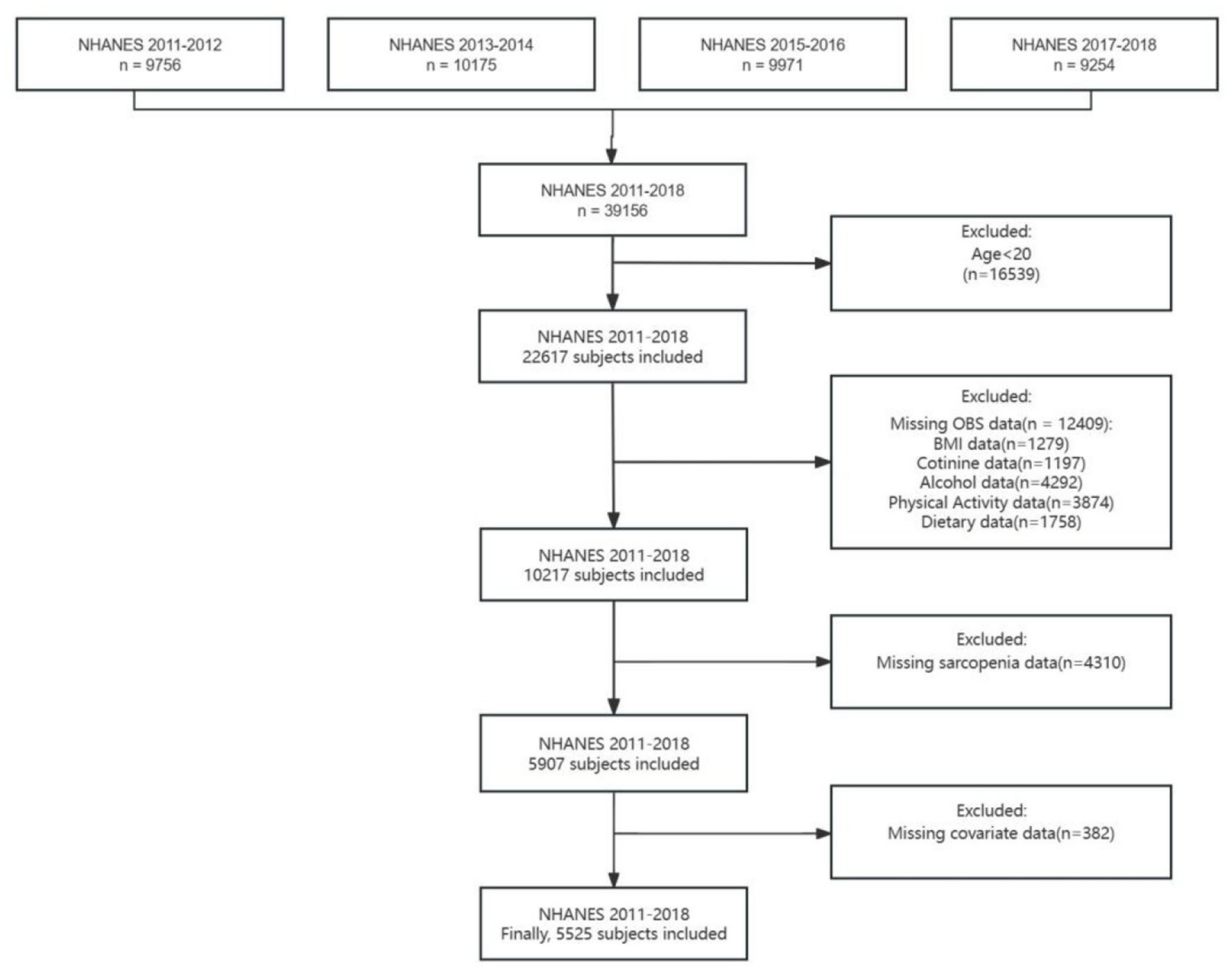
Flow chart of the sample selection from NHANES 2011–2018.

The details of our study's inclusion and exclusion criteria are represented in Figure 1. Among 39,156 participants in the four waves, some were excluded if (1) they were < 20 years of age (*n* = 16,539); (2) they had missing values concerning OBS data (*n* = 12,409)—including body mass index (BMI) (*n* = 1,279), cotinine (*n* = 1,197), alcohol use (*n* = 4,292), physical examination results (*n* = 3,874), and dietary data (*n* = 1,758); (3) they had missing sarcopenia data (*n* = 4,310); and (4) they had missing data on covariates (*n* = 382). Finally, 5,525 participants were enrolled in this study (Figure 1).

### 2.2 Assessment of sarcopenia

Sarcopenia was evaluated based on appendicular skeletal muscle mass (ASM). ASM was defined as the total sum of the lean soft tissue mass in the limbs, measured using dual-energy x-ray absorptiometry. A sarcopenia index of < 0.789 for men and < 0.512 for women was considered to indicate sarcopenia according to the recommendations published by the Foundation for National Institutes of Health Osteoarthritis Biomarkers study (FNIH). The sarcopenia index was calculated according to the following equation:

Sarcopenia index = total appendicular skeletal muscle mass/BMI (kg/m^2^) (19).

Based on the NHANES data, participants aged 8–59 years were eligible for dual-energy x-ray absorptiometry between 2011 and 2018.

### 2.3 Oxidative balance score

The calculation of the OBS, initially developed by Zhang et al. (14), was composed of 16 dietary and 4 lifestyle scores, including 5 oxidants and 15 antioxidants. An analysis of the mean values of two 24-h dietary review interviews provided dietary intake values for 16 nutrients. BMI, alcohol consumption, smoking status, and physical activity represent the elements of the lifestyle OBS. All of these components, except alcohol consumption, were categorized into sex-specific tertiles for this study. In our analysis of antioxidants, scores of 0, 1, and 2 were assigned from the lowest to the highest tertiles, whereas oxidants were ranked from 2 to 0. For alcohol consumption, we assigned 0 points to men who consumed >30 g/day and women who consumed >15 g/day; 1 point to men who consumed ≤ 30 g/day and women who consumed ≤ 15 g/day; and 2 points to non-drinkers. A higher total OBS score (i.e., the sum of all of the component scores) was considered to indicate a favorable oxidative balance. Supplementary Table 1 lists the classifications and assigned scores for each OBS factor.

### 2.4 Assessment of covariates

Our covariates for this study included demographic variables, dietary factors, and comorbidities. Demographic variables comprised sex, age group (young adults, 20–39 years; middle-aged adults, 40–59 years), ethnicity, educational level, and family income level (measured as family income to poverty [PIR]—with low family income, PIR ≤ 1.3; moderate family income, 1.3 < PIR < 3.5; and high family income, PIR ≥3.5). Total energy intake and chronic comorbidities—including diabetes, hypertension, and cardiovascular disease (CVD, including coronary heart disease, congestive heart failure, and angina)—were also assessed. Hypertension was determined by referring to a self-reported diagnosis by a doctor, use of antihypertensive medication, or high blood pressure (20). Diabetes was recorded if it had been diagnosed by a physician, if a participant was taking antidiabetic medication, had abnormal blood glucose, or if it was indicated in the glucose tolerance test results (21).

### 2.5 Statistical analysis

In light of the sample weighting selection guidelines of the NHANES, our study used the sampling weights recorded on dietary 2-day sample weight (WTDR2D) records. These records were divided by 4 since the survey had four consecutive cycles to ensure that our results were representative of the national population. Continuous variables are represented as weighted means (standard errors), and categorical ones are represented as counts (weighted percentages). To test the characteristic differences of variables in the four OBS groups, the Rao–Scott chi-squared and Kruskal–Wallis tests were used for categorical and non-normally distributed continuous variables, respectively.

Weighted logistic regression analysis was used to evaluate the association between OBS and sarcopenia. Model 1 represented the unadjusted analysis, whereas Model 2 adjusted for age, sex, ethnicity, educational level, and family income level. Model 3 increased the adjustments for energy intake, and complications such as hypertension, CVD, and diabetes were adjusted based on Model 2. We then transformed continuous OBS scores into quartile-categorical ones, exploring trend tests (P for trend). A subgroup analysis was also performed to observe the consistency of outcomes based on baseline variables. The stability of the results was tested using a sensitivity analysis. To investigate possible non-linear connections between OBS and sarcopenia among individuals, a restricted cubic spline (RCS) analysis was performed. All analyses were performed using various packages included in R Studio version 4.3.2. Statistical significance was set at a *p* < 0.05.

## 3 Results

### 3.1 Baseline characteristics

Table 1 describes the baseline characteristics of the participants based on OBS quartiles. A total of 5,525 participants were included in our study, consisting of 2,945 men (weighted 53%) and 2,580 women (weighted 47%), most of whom were non-Hispanic Caucasian in ethnicity (65%). The weighted prevalence of sarcopenia was 5.8% across the overall US population. Compared to the lowest OBS quartile, the participants in the highest one were more likely to be non-Hispanic Caucasian and less likely to have diabetes, higher educational levels, higher family incomes, and higher total energy intakes. However, across the quartiles, there were no significant differences in terms of sex, age, hypertension, or CVD.

**Table 1:** Baseline patient characteristics by OBS quartiles.

**Characteristic**	**Overall, *N =* 5,525 (100%)**	**Q1, *N =* 1,529 (25%)**	**Q2, *N =* 1,372 (24%)**	**Q3, *N =* 1,431 (27%)**	**Q4, *N =* 1,193 (23%)**	***P*-value**
Age						0.4
Young adults	2,762 (50%)	783 (51%)	680 (50%)	702 (47%)	597 (51%)	
Middle adults	2,763 (50%)	746 (49%)	692 (50%)	729 (53%)	596 (49%)	
Sex						0.4
Male	2,945 (53%)	805 (52%)	745 (55%)	753 (54%)	642 (50%)	
Female	2,580 (47%)	724 (48%)	627 (45%)	678 (46%)	551 (50%)	
Race						< 0.001
Mexican American	734 (9.6%)	158 (9.0%)	180 (9.4%)	197 (9.4%)	199 (10%)	
Other Hispanic	492 (6.3%)	130 (6.3%)	112 (6.2%)	127 (5.5%)	123 (7.2%)	
Non-Hispanic white	2,252 (65%)	602 (62%)	558 (64%)	613 (68%)	479 (67%)	
Non-Hispanic Black	1,106 (9.6%)	421 (15%)	287 (10%)	227 (7.3%)	171 (5.7%)	
Other/multiracial	941 (9.3%)	218 (8.1%)	235 (10%)	267 (9.5%)	221 (9.8%)	
Education level						< 0.001
> 9th Grade	172 (1.7%)	49 (1.7%)	45 (1.9%)	34 (1.1%)	44 (2.2%)	
9–11th Grade	572 (7.3%)	208 (11%)	144 (7.9%)	121 (5.6%)	99 (4.9%)	
High school Grad/GED	1,182 (21%)	411 (28%)	275 (22%)	295 (20%)	201 (12%)	
Some college or AA degree	1,894 (34%)	581 (40%)	490 (34%)	455 (30%)	368 (34%)	
College graduate or above	1,705 (36%)	280 (20%)	418 (33%)	526 (43%)	481 (47%)	
Poverty						< 0.001
Low family income	1,584 (21%)	542 (29%)	394 (21%)	360 (17%)	288 (16%)	
Middle family income	1,996 (33%)	581 (34%)	498 (34%)	508 (35%)	409 (28%)	
High family income	1,945 (46%)	406 (36%)	480 (44%)	563 (48%)	496 (56%)	
Total energy intake	2,186.96 (828.82)	1,585.36 (544.27)	2,006.39 (571.85)	2,360.53 (655.74)	2,818.17 (963.14)	< 0.001
Hypertension						0.064
Yes	1,505 (25%)	486 (28%)	363 (25%)	379 (25%)	277 (21%)	
No	4,020 (75%)	1,043 (72%)	1,009 (75%)	1,052 (75%)	916 (79%)	
Diabetes						0.002
Yes	511 (7.0%)	165 (9.4%)	136 (6.3%)	128 (7.9%)	82 (4.2%)	
No	5,014 (93%)	1,364 (91%)	1,236 (94%)	1,303 (92%)	1,111 (96%)	
Cardiovascular disease						0.2
Yes	105 (1.7%)	40 (2.0%)	24 (1.1%)	29 (2.4%)	12 (1.1%)	
No	5,420 (98%)	1,489 (98%)	1,348 (99%)	1,402 (98%)	1,181 (99%)	
Sarcopenia						< 0.001
Yes	379 (5.8%)	145 (9.3%)	96 (6.1%)	90 (5.4%)	48 (2.1%)	
No	5,146 (94%)	1,384 (91%)	1,276 (94%)	1,341 (95%)	1,145 (98%)	

### 3.2 Association between OBS and sarcopenia

The results of the weighted logistic regression analysis detailed in Table 2 indicate that sarcopenia was negatively associated with both continuous dietary OBS and OBS quartiles. In the fully adjusted model (Model 3), the second (OR: 0.62, 95% CI: 0.41, 0.94, *p* = 0.023), the third (OR: 0.50, 95% CI: 0.34, 0.74, *p* < 0.001), and highest quartiles (OR: 0.18, 95% CI: 0.11, 0.28, *p* < 0.001) of OBS were associated with higher risks of sarcopenia when compared to the lowest quartile. In our sensitivity analysis, the removal of any individual OBS component showed the same trend (*p* < 0.0001; Supplementary Table 2).

**Table 2:** Weighted logistic regression analysis models showing the associations between OBS and sarcopenia.

	**Model 1**			**Model 2**			**Model 3**		
**Characteristic**	**OR**	**95% CI**	***p*-value**	**OR**	**95% CI**	***p*-value**	**OR**	**95% CI**	***p-*value**
Continuous OBS	0.94	0.93, 0.96	< 0.001	0.94	0.93, 0.96	< 0.001	0.93	0.91, 0.95	< 0.001
Q1	Ref	Ref	Ref	Ref	Ref	Ref	Ref	Ref	Ref
Q2	0.63	0.43, 0.94	0.025	0.63	0.42, 0.95	0.027	0.62	0.41, 0.94	0.023
Q3	0.55	0.39, 0.78	0.001	0.57	0.41, 0.81	0.002	0.50	0.34, 0.74	< 0.001
Q4	0.21	0.14, 0.31	< 0.001	0.21	0.14, 0.31	< 0.001	0.18	0.11, 0.28	< 0.001
P for trend			< 0.001			< 0.001			< 0.001

OR, odds ratio; CI, confidence intervals. Model 1: unadjusted for age, sex, race, education level, and poverty. Model 2: adjusted for age, sex, race, education level, and poverty. Model 3: adjusted for total energy intake, hypertension, diabetes, and CVDs.

### 3.3 Association between dietary OBS, lifestyle OBS, and sarcopenia

Our study categorized OBS into dietary and lifestyle categories. The associations between dietary and lifestyle OBS with sarcopenia were evaluated separately (Table 3). Sarcopenia was negatively associated with continuous dietary OBS in a way that was statistically significant after adjusting for all covariates (OR = 0.94 [0.92, 0.97], *p* < 0.001). For lifestyle OBS, a higher score was strongly associated with a lower risk of sarcopenia after adjusting for all covariates (OR = 0.73 [0.66, 0.81], *p* < 0.001). Sarcopenia was equally affected by the dietary and lifestyle OBS quartiles. Lifestyle OBS affected sarcopenia more than dietary OBS.

**Table 3:** OR estimates for associations between dietary or lifestyle OBS and sarcopenia.

	**Model 1**			**Model 2**			**Model 3**		
**Characteristic**	**OR**	**95% CI**	***p*** **-value**	**OR**	**95% CI**	***p*** **-value**	**OR**	**95% CI**	***p*** **-value**
Continuous dietary OBS	0.95	0.94, 0.97	< 0.001	0.95	0.94, 0.97	< 0.001	0.94	0.92, 0.97	< 0.001
**Dietary OBS group**									
Q1	Ref	Ref	Ref	Ref	Ref	Ref	Ref	Ref	Ref
Q2	0.64	0.43, 0.96	0.031	0.65	0.43, 0.98	0.039	0.60	0.38, 0.95	0.031
Q3	0.59	0.41, 0.86	0.007	0.63	0.42, 0.93	0.022	0.56	0.36, 0.87	0.012
Q4	0.29	0.18, 0.47	< 0.001	0.29	0.18, 0.46	< 0.001	0.24	0.12, 0.48	< 0.001
P for trend			< 0.001			< 0.001			< 0.001
Continuous lifestyle OBS	0.69	0.63, 0.76	< 0.001	0.70	0.63, 0.77	< 0.001	0.73	0.66, 0.81	< 0.001
**Lifestyle OBS group**									
Q1	Ref	Ref	Ref	Ref	Ref	Ref	Ref	Ref	Ref
Q2	0.41	0.27, 0.61	< 0.001	0.42	0.28, 0.63	< 0.001	0.46	0.30, 0.71	< 0.001
Q3	0.31	0.19, 0.51	< 0.001	0.34	0.21, 0.57	< 0.001	0.39	0.23, 0.65	< 0.001
Q4	0.21	0.10, 0.46	< 0.001	0.22	0.10, 0.49	< 0.001	0.24	0.11, 0.56	0.001
P for trend			< 0.001			< 0.001			< 0.001

OR, odds ratio; CI, confidence intervals. Model 1: unadjusted for age, sex, race, education level, and poverty. Model 2: adjusted for age, sex, race, education level, and poverty. Model 3: adjusted for total energy intake, hypertension, diabetes, and cardiovascular disease.

### 3.4 Subgroup analyses and interaction effects of the association between OBS and sarcopenia

**Figure 2:**
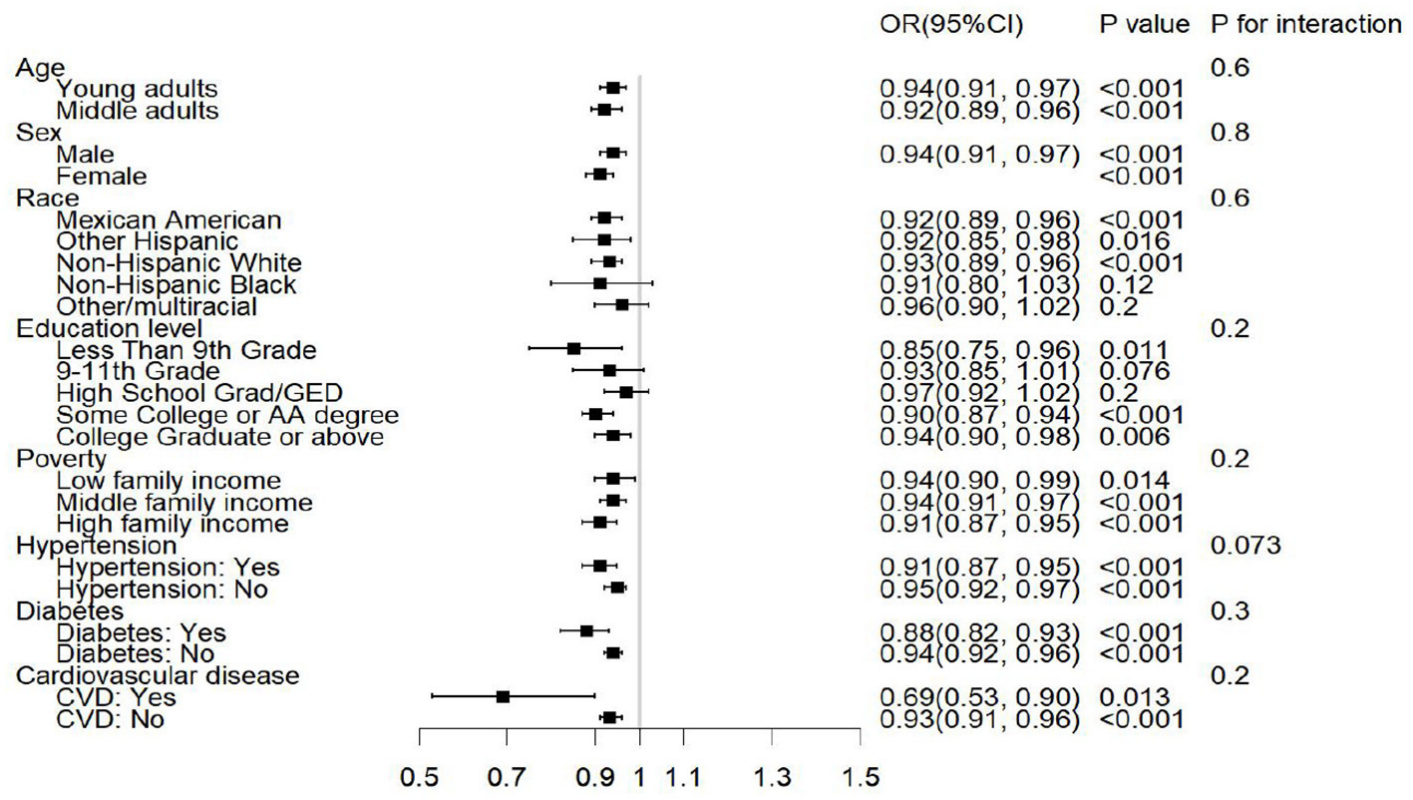
Forest plot for our subgroup analyses. P value: adjusted *P* value for Model 3.

Through a subgroup analysis, the association between OBS and sarcopenia was explored by considering age, sex, ethnicity, educational level, family income, and comorbidities such as diabetes, hypertension, and CVD—as is shown in Figure 2. Higher OBS is associated with a lower risk of sarcopenia in subgroups stratified by age, sex, family income, and presence of diabetes, hypertension, or CVD (P for trend, < 0.05). This subgroup analysis showed that the CVD subgroup was more sensitive to OBS than the non-CVD one. Individuals with CVD had a 34% lower risk of developing sarcopenia with higher OBS scores. By contrast, the risk of sarcopenia only decreased by 7% in those individuals without CVD. Non-Hispanic African–Americans, people of other ethnicities, and those with high school levels of education did not show significant associations with sarcopenia. No significant differences were observed between the subgroups of the covariates in our interaction analysis (Figure 2).

### 3.5 Restricted cubic spline analysis

**Figure 3:**
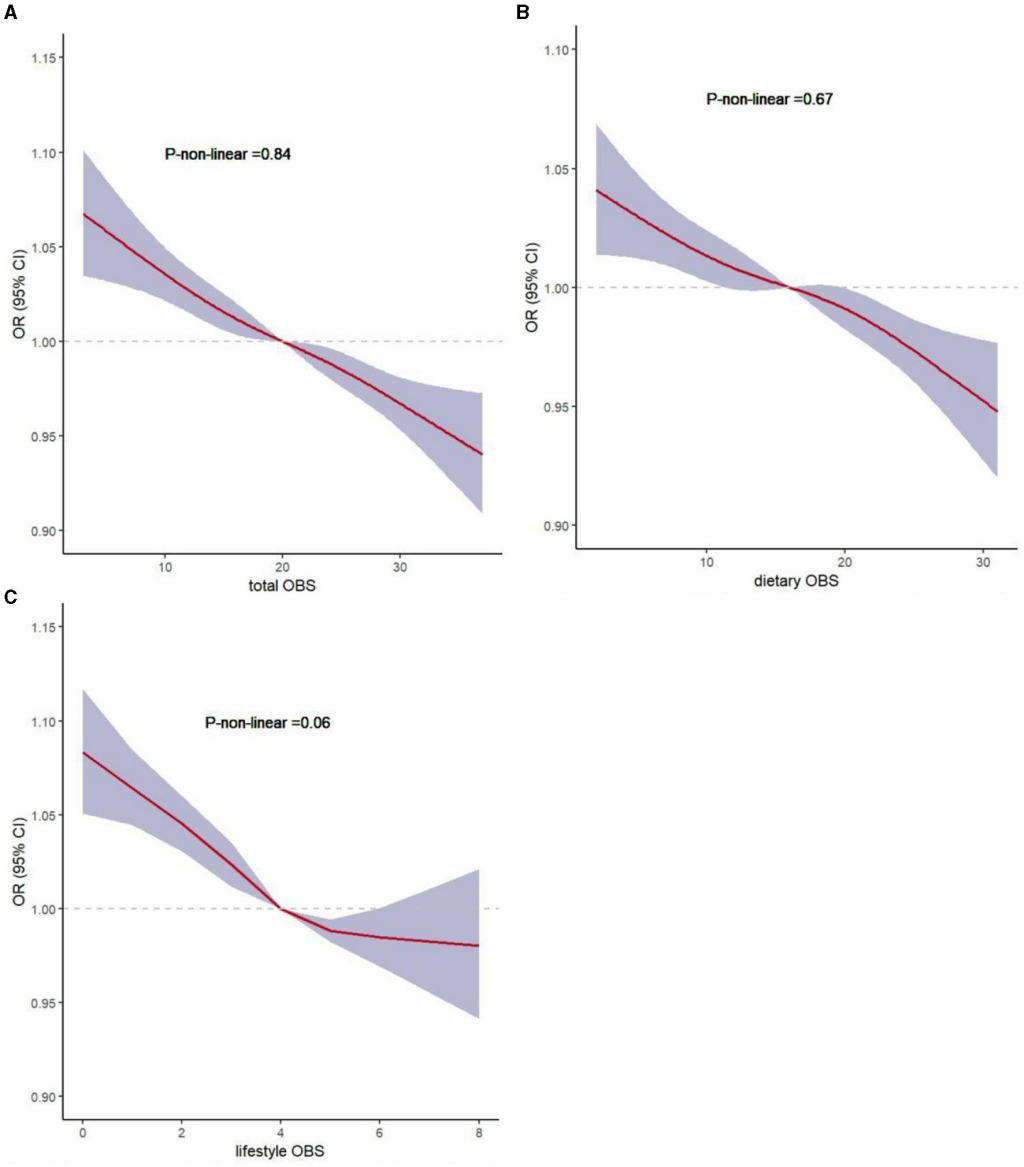
Non-linear associations of total OBS, dietary OBS, and lifestyle OBS with sarcopenia by restricted cubic spline analysis. **(A)** total OBS and sarcopenia; **(B)** dietary OBS and sarcopenia; **(C)** lifestyle OBS and sarcopenia.

To evaluate the non-linear associations between OBS, dietary OBS, lifestyle OBS, and sarcopenia, we performed a restricted cubic spline (RCS) analysis based on the weighted logistic regression adjusted for Model 3. Figures 3A–C show that the non-linear represented p was >0.05, indicating a linear association between these variables.

## 4 Discussion

Using data from NHANES 2011–2018, we conducted a cross-sectional study to clarify the negative association between OBS and the risk of sarcopenia, demonstrating that a higher OBS may be associated with a lower risk of sarcopenia. Despite adjusting for possible confounders, this association remained significant for both dietary and lifestyle OBS.

Sarcopenia-associated skeletal muscle loss generally begins at the age of 35 years and progresses at a rate of 1%−2% per year (22). Recent studies have revealed that pre-sarcopenia was identified in 33%−66% of healthy young women in Japan (23, 24). Therefore, it is imperative to initiate preventive measures against frailty at a young age to circumvent potential future mobility challenges or conditions such as sarcopenia.

The multifactorial and intricate pathogenesis of sarcopenia can be caused by a range of factors including genetics, aging, diet, and physical inactivity (25). Skeletal muscle loss has been linked to oxidative stress, inflammation, insulin resistance, mitochondrial dysfunction, and telomere shortening, among other factors (12, 26–30). The skeletal muscle becomes more vulnerable to oxidative stress with increase in metabolic activity, resulting in ROS production (31). Oxidative stress is increased in sarcopenia, leading to an increased risk of CVD in sarcopenic obesity and chronic obstructive pulmonary disease (32, 33). Bernabeu-Wittel et al. reported that oxidative stress markers, including lower total antioxidant capacity for ROS (TAC-ROS) and higher superoxide dismutase (SOD) levels, were increased in sarcopenia patients (34). Oxidants in tobacco smoke and obesity can cause oxidative stress by producing ROS or reducing the defensive mechanisms of the antioxidant system, including several dietary components (35). Individuals with antioxidant deficiency coupled with poor nutrition are more likely to experience adverse health reactions as a result of oxidative stress (36). Therefore, it is a feasible strategy for individuals at high risk of sarcopenia to reduce free radical generation through antioxidant treatments. ROS can be produced either by endogenous antioxidant defense systems or by exogenous systems (i.e., dietary intake) (37). The OBS is a synthetic metric used to assess the overall exposure balance of dietary and lifestyle oxidants and antioxidants (14). The results of our study support the current understanding of the role of OS in the pathogenesis of sarcopenia, as a higher OBS level protects against sarcopenia.

Although no direct evidence has been reported linking OBS with sarcopenia, several studies have demonstrated an association between dietary intake and sarcopenia. A higher intake of fiber may result in a greater abundance of specific gut bacteria, which slows the development of sarcopenia (38). Vitamins C and E are antioxidant vitamins, as they can scavenge ROS and promote cellular antioxidant activity (39). A study by Lewis et al. reported a positive association between dietary and circulating vitamin C levels and skeletal muscle mass in middle-aged and older individuals (40). Vitamin E plays a significant role in the recovery of myogenic cell membranes and is beneficial for treating muscle cells (41). One cross-sectional study showed that dietary and plasma vitamin E concentrations can reduce the risk of both hip and total fractures (42). Intake of a wide range of B vitamins can also reduce the risk of sarcopenia. Niacin, also known as vitamin B3, plays a vital role in preventing sarcopenia (43). Moreover, consumption of B6 may be beneficial in combating sarcopenia, frailty, and all-cause mortality (44). During B6 deficiency, the modulation of PLP-dependent enzymes results in several pathophysiological states that can impair skeletal muscle function (44). Sufficient evidence indicates that low micronutrient intake can result in a higher risk of sarcopenia. Magnesium is an important cofactor in many enzymatic reactions essential for the preservation of muscle mass and the protection of muscle tissue from oxidative damage (45, 46). In a cross-sectional study, oral intake of magnesium was observed to potentially prevent sarcopenia (47). One systematic review reported that sarcopenia could be prevented and treated by increasing intake of micronutrients such as calcium, iron, magnesium, phosphorus, potassium, selenium, sodium, and zinc (48). The benefits of dietary antioxidants, including the reduction of oxidative stress in skeletal muscles, are well known (49). The Composite Dietary Antioxidant Index (CDAI), which includes vitamins A, C, and E; carotenoids; zinc; and selenium, was examined in a recent study (49). The CDAI is related to hand grip strength, which indirectly supports the evidence that dietary factors contribute to sarcopenia (49).

The preservation, loss, and restoration of muscle mass rely on the balance between two concurrent yet opposing processes: protein degradation (PD) and protein synthesis (PS), collectively referred to as protein turnover. (50). The mTOR kinase serves as a central regulator of muscle protein synthesis, and its activity is significantly influenced by the availability of nutrients—particularly amino acids and growth factors (50). Whey proteins may further enhance antioxidant capacity by providing cysteine-rich proteins, which work in the synthesis of glutathione, a crucial intracellular antioxidant (51).

A correlation between a healthy lifestyle and sarcopenia has been confirmed. To date, physical activity has been proven to help maintain muscle health and delay sarcopenia (52), and resistance exercise, in particular, is a potent stimulus for PS (53). In the absence of exercise, muscle atrophy occurs because of reduced production of myocytokines, an imbalance between anti-inflammatory and pro-inflammatory mediators, and altered protein synthesis (54). However, consistent research has demonstrated that exercise generates ROS, which can be both beneficial or harmful depending on several factors, including the concentration of ROS, the duration of exposure, and the individual's training status (55).For example, ROS triggers the mitochondrial biogenesis cascade in response to endurance exercise (56). Additionally, within physiological concentrations, ROS regulates antioxidant systems by boosting the content and activities of SOD1 and SOD2 in muscle cells, thereby reducing ROS concentration (57).Given the current findings regarding oxidative balance and sarcopenia, it could be hypothesized that the impact of exercise on sarcopenia is multifaceted and may involve the regulation of oxidative balance. Further research is needed to prove this hypothesis.

Tobacco smoking and alcohol consumption represent two other primary factors that can affect muscle health. The effects of smoking include inactivity, lack of energy, and systemic inflammation—all of which interfere with muscle protein synthesis (58). A longitudinal cohort study investigating the correlation between smoking and the prevalence of sarcopenia observed that smokers had a 2.36-fold higher risk of developing sarcopenia than non-smokers (59). The prevalence of sarcopenia was aggravated by excessive alcohol consumption, possibly because skeletal muscle protein synthesis is restricted by excessive alcohol consumption (58, 60). BMI was used to assess body fat and thickness in this study. A previous study discovered an association between abdominal obesity and muscle mass and strength decline (61). Some individuals have both obesity and sarcopenia, sometimes referred to as sarcopenic obesity. The systemic energy burden associated with obesity accelerates disease onset by driving muscle loss (62).

These results further demonstrate the importance of antioxidant capacity in patients with sarcopenia or those at risk of developing it. Thus, we measured the oxidative balance in our participants and assessed its role in sarcopenia using OBS—a comprehensive indicator. Our study indicated that OBS was significantly higher in the non-sarcopenia group than in the sarcopenia one. Furthermore, observation of continuous OBS showed that lifestyle OBS had a more positive influence on sarcopenia than dietary OBS. After stratification by age, sex, ethnicity, educational level, family income, hypertension, diabetes, and CVD, no substantial alterations were observed in the association between OBS and sarcopenia.

Our study had several notable strengths. First, we used data from a nationally representative population-based study to generate our results, making them more generalizable. Furthermore, OBS, as a comprehensive measurement method, provides an assessment of multiple aspects of oxidant–antioxidant exposure. Finally, as opposed to older adults, our study surveyed the relationship between OBS and sarcopenia in young and middle-aged adults. However, this study also had several key limitations. Owing to its cross-sectional design, we were unable to establish a causal relationship between OBS and sarcopenia. Second, sarcopenia is defined as a decline in skeletal muscle mass with functional deterioration. In this study, sarcopenia was assessed based on low muscle mass. In the NHANES datasets, inadequate information regarding muscle function was found. Thus, future studies for determining the correlation between muscle function and OBS should be conducted. In addition, collecting 24-h dietary data may induce recall bias and fail to reflect long-term dietary patterns. Therefore, further clinical randomized controlled trials are warranted to confirm our findings.

## 5 Conclusion

This study showed that higher antioxidant and lower oxidant exposure may decrease the risk of developing sarcopenia. A higher OBS may indicate higher protection against sarcopenia. Therefore, individuals may be able to reduce the loss of muscle mass and delay the development of sarcopenia by maintaining healthy lifestyles and taking dietary antioxidant supplements. However, further clinical studies are warranted to confirm these findings.

## Data availability statement

Publicly available datasets were analyzed in this study. This data can be found here: https://wwwn.cdc.gov/nchs/nhanes.

## Ethics statement

The studies involving humans were approved by the National Center for Health Statistics' Ethics Review Board. The studies were conducted in accordance with the local legislation and institutional requirements. The participants provided their written informed consent to participate in this study.

## Author contributions

ZC: Formal analysis, Methodology, Supervision, Writing – original draft, Writing – review & editing. DD: Formal analysis, Writing – review & editing.
