# Supplementary material for: Association of the oxidative balance score with sarcopenia among young and middle-aged adults: findings from NHANES 2011–2018
Source: Front Nutr. 2024 Jun 4;11:1397429. doi: 10.3389/fnut.2024.1397429 (PMC11183506; doi:10.3389/fnut.2024.1397429)
Supplement: Supplementary file 1 [file Table_1.docx]

Supplementary Table 1. Ingredients that make up the oxidative balance score.

| OBS components | Property | Male |  |  | Female |  |  |
| --- | --- | --- | --- | --- | --- | --- | --- |
|  |  | 0 | 1 | 2 | 0 | 1 | 2 |
| **Lifestyle OBS components** |  |  |  |  |  |  |  |
| Physical activity (MET-minute/week) | A | ≤1920 | 1920-6600 | ＞6600 | ≤1200 | 1200-3480 | ＞3480 |
| Alcohol (g/d) | P | ＞30 | 0-30 | 0 | ＜15 | 0-15 | 0 |
| Body mass index (kg/m2) | P | ＞29.9 | 25.5-29.9 | ≤25.5 | ＜31.4 | 24.9-31.4 | ≤24.9 |
| Cotinine (ng/mL) | P | ＞12.7 | 0.024-12.7 | ≤0.024 | ＜0.158 | 0.015-0.158 | ≤0.015 |
| **Dietary OBS components** |  |  |  |  |  |  |  |
| Dietary fiber (g/d) | A | ≤13.4 | 13.4-21.8 | ＞21.8 | ≤11.3 | 11.3-17.6 | ＞17.6 |
| β -Carotene (RE/d) | A | ≤586 | 586-1884 | ＞1884 | ≤630 | 627-2123 | ＞2128 |
| Riboflavin (mg/d) | A | ≤1.8 | 1.8-2.64 | ＞2.64 | ≤1.39 | 1.39-2 | ＞2 |
| Niacin (mg/d) | A | ≤25.093 | 25.093-35.054 | ＞35.054 | ≤17.235 | 17.235-24.151 | ＞24.151 |
| Vitamin B6 (mg/d) | A | ≤1.862 | 1.862-2.753 | ＞2.753 | ≤1.319 | 1.319-1.960 | ＞1.960 |
| Total folate (mcg/d) | A | ≤342 | 342-515 | ＞515 | ≤259 | 259-386 | ＞386 |
| Vitamin B12 (mcg/d) | A | ≤3.65 | 3.65-6.31 | ＞6.31 | ≤2.5 | 2.5-4.35 | ＞4.35 |
| Vitamin C (mg/d) | A | ≤39.5 | 39.5-99 | ＞99 | ≤37 | 37-84.4 | ＞84.4 |
| Vitamin E (ATE) (mg/d) | A | ≤6.73 | 6.73-10.6 | ＞10.6 | ≤5.76 | 5.76-8.90 | ＞8.90 |
| Calcium (mg/d) | A | ≤800 | 800-1210 | ＞1210 | ≤631 | 631-958 | ＞958 |
| Magnesium (mg/d) | A | ≤271.167 | 271.167-387.33 | ＞387.33 | ≤214.5 | 214.5-296.66 | ＞296.66 |
| Zinc (mg/d) | A | ≤10.185 | 10.185-14.715 | ＞14.715 | ≤7.305 | 7.305-10.423 | ＞10.423 |
| Copper (mg/d) | A | ≤1.043 | 1.043-1.513 | ＞1.513 | ≤0.846 | 0.836-1.212 | ＞1.212 |
| Selenium (mcg/d) | A | ≤111 | 111-155 | ＞155 | ≤79 | 79-112 | ＞112 |
| Total fat (g/d) | P | ＞106 | 73.9-106 | ≤73.9 | ＞80 | 56.3-80 | ≤56.3 |
| Iron (mg/d) | A | ≤12.775 | 12.775-18.25 | ＞18.25 | ≤9.53 | 9.53-13.72 | ＞13.72 |

A stood in for the antioxidant, P for the pro-oxidant. RE for the retinal equivalent, ATE for the alpha-tocopherol equivalent, and MET for the metabolic equivalent.

Supplementary Table 2: Sensitivity analyses to evaluate the effects of individual OBS components on the sarcopenia.

| **OBS** | **Sarcopenia** | |  |
| --- | --- | --- | --- |
| **OBS adjusted for model 3** | **OR** | **95% CI** | **p-value** |
| 1OBS excluding physical activity | 0.93 | 0.91, 0.95 | <0.001 |
| 2OBS excluding alcohol | 0.93 | 0.91, 0.95 | <0.001 |
| 3OBS excluding body mass index | 0.95 | 0.92, 0.97 | <0.001 |
| 4OBS excluding cotinine | 0.93 | 0.90, 0.95 | <0.001 |
| 5OBS excluding dietary fiber | 0.93 | 0.90, 0.95 | <0.001 |
| 6OBS excluding carotene | 0.93 | 0.91, 0.95 | <0.001 |
| 7OBS excluding riboflavin | 0.93 | 0.90, 0.95 | <0.001 |
| 8OBS excluding niacin | 0.93 | 0.90, 0.95 | <0.001 |
| 9OBS excluding vitamin b6 | 0.93 | 0.90, 0.95 | <0.001 |
| 10OBS excluding total folate | 0.92 | 0.90, 0.95 | <0.001 |
| 11OBS excluding vitamin b12 | 0.92 | 0.90, 0.94 | <0.001 |
| 12OBS excluding vitamin c | 0.93 | 0.90, 0.95 | <0.001 |
| 13OBS excluding vitamin e | 0.93 | 0.90, 0.95 | <0.001 |
| 14OBS excluding calcium | 0.93 | 0.91, 0.95 | <0.001 |
| 15OBS excluding magnesium | 0.93 | 0.90, 0.95 | <0.001 |
| 16OBS excluding zinc | 0.92 | 0.90, 0.95 | <0.001 |
| 17OBS excluding copper | 0.93 | 0.90, 0.95 | <0.001 |
| 18OBS excluding selenium | 0.92 | 0.90, 0.95 | <0.001 |
| 19OBS excluding total fat | 0.93 | 0.91, 0.95 | <0.001 |
| 20OBS excluding iron | 0.92 | 0.90, 0.94 | <0.001 |

OR, odds ratio; CI, confidence intervals
